# Eye movements in real and simulated driving and navigation control – Foreword to the Special Issue

**DOI:** 10.16910/jemr.12.3.0

**Published:** 2021-06-03

**Authors:** Rudolf Groner, Enkelejda Kasneci

**Affiliations:** scians Ltd, Bern, Switzerland; www.scians.ch; University of Tübingen, Tübingen, Germany

**Keywords:** Eye movement, eye tracking, driving, navigation control, simulator, human operator, cognitive load, autonomous driving, eye computer interaction, ECI, cognitive load, situational awareness

## Abstract

The control of technological systems by human operators has been the object of study for many decades. The increasing complexity in the digital age has made the optimization of the interaction between system and human operator particularly necessary. In the present thematic issue, ten exemplary articles are presented, ranging from observational field studies to experimental work in highly complex navigation simulators. For the human operator, the processes of attention play a crucial role, which are captured in the contributions listed in this thematic issue by eye-tracking devices.

For many decades, eye tracking during car driving has been investigated extensively (e.g. 6; 5). In the present special issue, Cvahte Ojsteršek & Topolšek (4) provide a literature review and scientometric analysis of 139 eye-tracking studies investigating driver distraction. For future studies, the authors recommend a wider variety of distractor stimuli, a larger number of tested participants, and an increasing interdisciplinarity of researchers.

In addition to most studies investigating bottom-up processes of covered attention, Tuhkanen, Pekkanen, Lehtonen & Lappi (10) include the experimental control of top-down processes of overt attention in an active visuomotor steering task. The results indicate a bottom-up process of biasing the optic flow of the stimulus input in interaction with the top-down saccade planning induced by the steering task.

An expanding area of technological development involves autonomous driving where actions of the human operator directly interact with the programmed reactions of the vehicle. Autonomous driving requires, however, a broader exploration of the entire visual input and less gaze directed towards the road centre. Schnebelen, Charron & Mars (9) conducted experimental research in this area and concluded that gaze dynamics played the most important role in distinguishing between manual and automated driving.

Through a combination of advanced gaze tracking systems with the latest vehicle environment sensors, Bickerdt, Wendland, Geisler, Sonnenberg & Kasneci (2021) conducted a study with 50 participants in a driving simulator and propose a novel way to determine perceptual limits which are applicable to realistic driving scenarios.

Eye-Computer-Interaction (ECI) is an interactive method of directly controlling a technological device by means of ocular parameters. In this context, Niu, Gao, Xue, Zhang & Yang (8) conducted two experiments to explore the optimum target size and gaze-triggering dwell time in ECI. Their results have an exemplary application value for future interface design.

Aircraft training and pilot selection is commonly performed on simulators. This makes it possible to study human capabilities and their limitation in interaction with the simulated technological system. Based on their methodological developments and experimental results, Vlačić, Knežević, Mandal, Rođenkov & Vitsas (11) propose a network approach with three target measures describing the individual saccade strategy of the participants in this study.

In their analysis of the cognitive load of pilots, Babu, Jeevitha Shree, Prabhakar, Saluja, Pashilkar & Biswas (3) investigated the ocular parameters of 14 pilots in a simulator and during test flights in an aircraft during air to ground attack training. Their results showed that ocular parameters are significantly different in different flying conditions and significantly correlate with altitude gradients during air to ground dive training tasks.

In maritime training the use of simulations is per international regulations mandatory. Mao, Hildre & Zhang (7) performed a study of crane lifting and compared novice and expert operators. Similarities and dissimilarities of eye behavior between novice and expert are outlined and discussed.

The study of Atik & Arslan (2) involves capturing and analyzing eye movement data of ship officers with sea experience in simulation exercises for assessing competency. Significant differences were found between electronic navigation competencies of expert and novice ship officers. The authors demonstrate that the eye tracking technology is a valuable tool for the assessment of electronic navigation competency.

The focus of the study by Atik (1) is the assessment and training of situational awareness of ship officers in naval Bridge Resource Management. The study shows that eye tracking provides the assessor with important novel data in simulator based maritime training, such as focus of attention, which is a decisive factor for the effectiveness of Bridge Resource Management training.

The research presented in the different articles of this special thematic issue cover many different areas of application and involve specialists from different fields, but they converge on repeated demonstrations of the usefulness of measuring attentional processes by eye movements or using gaze parameters for controlling complex technological devices. Together, they share the common goal of improving the potential and safety of technology in the digital age by fitting it to human capabilities and limitations.
